# Chiasmal herniation following treatment of pituitary macroadenoma

**DOI:** 10.1007/s11102-020-01088-2

**Published:** 2020-10-15

**Authors:** Marjolein Tabak, Iris C. M. Pelsma, Mark C. Kruit, Wouter R. van Furth, Nienke R. Biermasz, Irene C. Notting

**Affiliations:** 1 Department of Ophthalmology, Leiden University Medical Center, Leiden, The Netherlands; 2 Department of Medicine, Division of Endocrinology, Leiden University Medical Center, Leiden, The Netherlands; 3 Department of Radiology, Leiden University Medical Center, Leiden, The Netherlands; 4 Department of Neurosurgery, Leiden University Medical Center, Leiden, The Netherlands; 5 Center for Pituitary Tumours Leiden, Leiden University Medical Center, Leiden, The Netherlands

**Keywords:** Pituitary adenoma, Optic chiasm, Chiasmal herniation, Visual field, Visual deterioration, Chiasmapexy

## Abstract

**Purpose:**

To evaluate whether the occurrence of chiasmal herniation coincides with visual field (VF) deterioration and to compare the course of VF defects in patients with and without radiological chiasmal herniation following treatment of pituitary adenoma.

**Methods:**

This retrospective cohort study included 48 pituitary macroadenoma patients with chiasm compression, divided into three groups: Group 1 (N = 12), downward displaced optic chiasm and deteriorated VFs; Group 2 (N = 16), downward displaced optic chiasm; Group 3 (N = 20), control-group matched for tumour size and follow-up VFs, in mean deviation (dB). VFs were compared over time and a severity index, Chiasm Herniation Scale (CHS), for herniation based on radiological parameters was designed.

**Results:**

After treatment, all groups showed improvement of VFs (Gr1: 2.97 dB p = 0.097, Gr2: 4.52 dB p = 0.001 and Gr3: 5.16 dB p = 0.000), followed by long-term gradual deterioration. The course of VFs between patients with and without herniation was not significantly different (p = 0.143), neither was there a difference in the course before and after herniation (p = 0.297). The median time till onset of herniation was 40 months (IQR 6 month-10 years) and did not significantly differ (p = 0.172) between the groups. There was no relation between VFs and the degree of herniation (p = 0.729).

**Conclusion:**

Herniation does not appear to have clinical relevance with respect to VF outcome. The newly designed CHS is the first scoring system to quantify the severity of herniation and, in the absence of alternatives, may be useful to describe MRI findings to serve future added value in larger sized outcome studies.

## Introduction

Patients with pituitary macroadenomas typically present with bitemporal hemianopsia, a partial blindness where vision is missing in the outer half of the visual field (VF), as a result of an elevated and compressed optic chiasm [1–3]. Based on the type of tumour, decompression by resection or size reduction by pharmacological treatment results in immediate and significant improvement of VF in the vast majority of patients [4–8]. However, in some patients, delayed deterioration of the VF and visual acuity (VA) is observed following initial post-treatment improvement. In the clinical work-up of this deterioration, a Magnetic Resonance Imaging (MRI) scan is performed to exclude new chiasmal compression caused by a recurrent tumour or growth of a remnant. Strikingly, several of these patients showed a radiological herniation, i.e. a (new) downward displacement of the optic chiasm into a secondary enlarged sella, raising the question whether this is (one of) the reasons for deterioration of VF [2, 9, 10].

The most accepted hypotheses regarding the pathophysiological mechanism of the secondary downward displacement of the optic chiasm and its relation with deteriorating VF and VA is based on tethering of scar tissue, enlargement of the sella turcica and a deficient sellar diaphragm, the roof of the sella turcica or a combination. This results in downward displacement of the optic chiasm, stretching the optic nerves and causing deterioration of the VF [10–12].

A PubMed search resulted in a total of 23 studies, and most studies focusing on downward displacement of the optic chiasm and VF deterioration were case reports. Between 1968 and 2019, 45 cases were described with visual deterioration after pituitary adenoma treatment, were the MRI showed a radiological herniation of the optic chiasm. The incidence of visual deterioration and herniation of the optic chiasm varied from 0.8 to 10% (4 out of 501 and 3 out of 28) [9, 13]. Patients were aged between 20 and 71 years, 55% had an endocrine active adenoma, 43% had a nonfunctioning pituitary adenoma (NFA) and 2% a Rathke’s Cleft Cyst (RCC) [2, 9, 10, 13–30]. Furthermore, in 8 patients with secondary empty sella, chiasmal herniation and the severity of visual symptoms were not related [10].

The reduction of or withdrawal from dopamine-agonists improved VA in spite of the remaining downward displacement in prolactinoma patients [2, 13, 22, 27, 30]. Another method of resolving the downward displacement of the optic chiasm was chiasmapexy—literally meaning ‘fixing the chiasm’—a surgical technique to elevate the optic chiasm. In some cases, VF and VA were reportedly improved following chiasmapexy [9, 12, 14, 16–20, 23–25, 29, 31]. By contrast, in two patients VF remained stable over > 1 year and in one case spontaneous improvement of VF was seen following watchful waiting [18, 26, 32]. Therefore, chiasmapexy is a procedure with unknown efficacy, since the natural course of VF and VA following herniation is unknown and no influencing factors have been identified yet.

This study aimed to compare the natural course of VF, obtained with (perimetric) VF tests and depicted as mean deviation (MD) in dB, in patients with and without herniation of the optic chiasm, and to assess whether herniation of the optic chiasm and VF deterioration were (causally) related. Patients were divided in three groups: Group 1, with downward displaced optic chiasm followed at the Ophthalmology department for deteriorated VF. Group 2, with only downward displaced optic chiasm primarily observed on the MRI. Group 3, the control-group with identical baseline tumour characteristics. In these three cohorts, VF and VF deterioration were compared over time to identify the relation between herniation of the optic chiasm and VF.

## Materials and methods

The design of the study is an observational, retrospective longitudinal cohort study. The study is approved by the Scientific Committee of the Department Ophthalmology of the Leiden University Medical Center (LUMC). Informed consent and approval by Medical Ethical Validation Committee was waived.

### Patients

Included patients had a history of treated chiasm-compressing pituitary macroadenoma causing a VF deficit and were older than 18 years. All patients were treated using one, or a combination, of the widely accepted treatment modalities (e.g. surgical, pharmacological or radiotherapy). Because glaucoma, a disease with a raised intraocular pressure (IOP) can result in typical VF defects, patients with a history of glaucoma or otherwise raised IOP were discussed with an ophthalmologist to ensure evaluability of the VF. When IOP was under control and VF was atypical for glaucoma (defects not crossing the midline) during the follow-up period, patients were included for the study. Patients with a large adenoma with a cranial caudal tumour size of more than 50 mm were excluded since the postoperative anatomy of the pituitary, sinuses and optic pathways has been significantly altered to the extent that using the Chiasm Herniation Scale (CHS) was deemed impossible. Additional exclusion criteria were insufficient follow-up data, no treatment, tumour remnant with chiasmal compression, first treatment before 1980, death, no light perception or other ophthalmologic disease resulting in deteriorating VF.

Included patients were divided into three groups. Group 1 was selected via the Ophthalmology department, because of downward displacement of the optic chiasm and deteriorating VF resulting in more intense and prolonged ophthalmologic follow-up. Patients included in Group 2 and 3 were selected from a clinical database with pituitary adenoma patients of the LUMC. Patients were included in Group 2 in case of radiological signs of downward displacement of the optic chiasm with or without ophthalmological symptoms. Group 3, the control group, was included based on the absence of downward displacement of the optic chiasm and was matched on cranial caudal tumour size and follow-up time with Group 1.

### Study parameters

All included patients were treated and followed according to standard patient care based on the pituitary protocol of the LUMC. In summary, this protocol included at least 3 ophthalmological appointments with Humphrey Field Analyzer (HFA) perimetric VF assessment, radiological assessment using an MRI scan within 6 months postoperatively, and multiple appointments with a neurosurgeon and endocrinologist for clinical assessment. Radiological surveillance after 6 months was individualized, based on e.g. the presence of a remnant, and clinical symptoms.

All study parameters were obtained from electronic patient files. Basic characteristics collected were: sex, age, type of tumour, cranial caudal tumour size, Fujimoto score, compression of the optic chiasm, moment of treatment, type of treatment (surgery/radiotherapy/medication) and visual symptoms before treatment. All follow-up measurements of VA, VF and IOP, were collected separately for the right and left eye. In the next paragraphs, the different measurements, scales and denominators will be explained in detail.

### Visual function assessments

Because of the long follow-up of our patients, the utilization of newer generations of perimeters and newer software, we used different methods for perimetry. For all measurement modalities, the standard settings were used for VF analysis. Most measurements were performed using the HFA static perimetry for determining the MD in dB and, as of 2010/2011, Visual Field Index (VFI) in %. VF was analysed using the 30–2 threshold program, which measured VF in 30 degrees temporally and nasally by testing 76 points. The MD score depicts the VFs compared to a healthy control group. A recent literature study showed that this score can be used to assess the VF for patients with pituitary adenomas [33]. Unreliable measurements, based on loss of fixation, falls positive and falls negative measurements, were excluded, as reviewed by two independent observers (M.T., I.C.N). VA has been measured using the ‘Snellen’ chart, the standard distance acuity chart used in the Netherlands, measuring the vision on a scale from 0 to 2.0, with 1.0 being the mean in a healthy control group. The definition used for (clinically important) deterioration was more than 1 dB MD loss, more than 3% VFI loss or more than 0.1 point VA loss, in two successive measurements.

### Radiology

The tumours and the condition of the optic chiasm were assessed using MRI. In some cases, however, the first images were made using Computed Tomography (CT) scan to diagnose the tumour. In these cases, the cranial caudal tumour size as shown on the CT was measured. Other measured factors, e.g. angle and thickness of the optic chiasm, downward displacement in mm, Fujimoto score and size of the sella could not be assessed on CT scans.

Firstly, the optic chiasm was classified as ‘compressed’, ‘normal’ or ‘downward displaced’ for inclusion (M.T.). Downward displacement was defined as a partial or total sellar location of the suprasellar visual system, which means that the chiasm is situated below the plane of the diaphragm sellae and shows a typical ‘V’-shape [10].

**Fig. 1:**
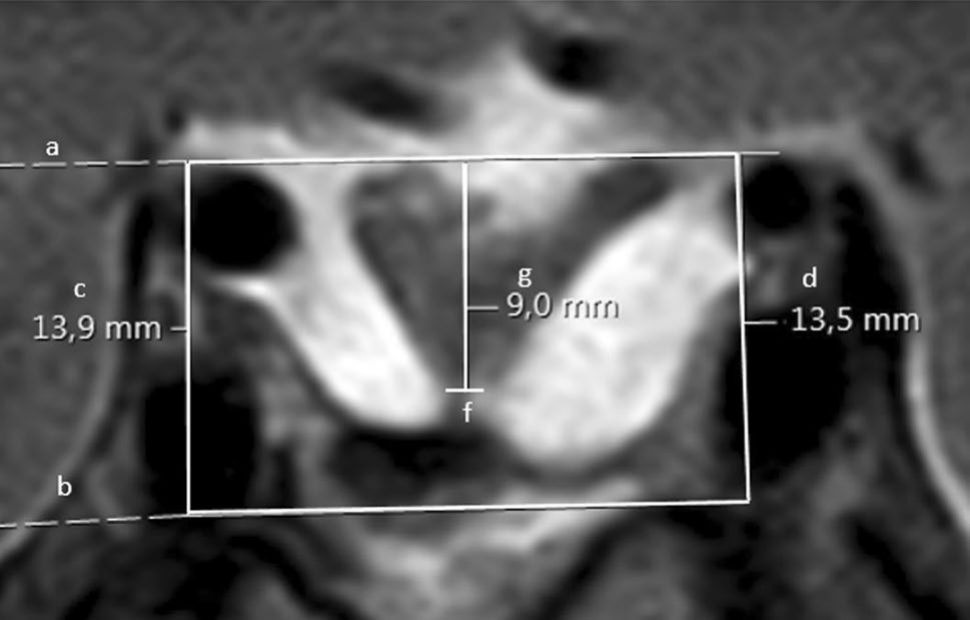
MRI scan demonstrating a downward displaced optic chiasm classified with the Chiasm Herniation Scale (CHS). Line (a) connects the superior border of the superior carotid arteries at their entry point into the cavernous sinus. Line (b) connects the inferior border of the inferior carotid arteries at their entry point into the cavernous sinus. The mean distance between those lines is (e) (e = 13.7 mm), extracted from (c) (c = 13.9 mm) and (d) (d = 13.5 mm). The lowest point of the optic chiasm is (f). The distance from (f) to line (a) is (g) (g = 9.0). The proportional herniation is calculated with (e) and (g). Example: ${\text{CHS }} = { 1}00 \, \times {\text{ g}}/{\text{e}}\quad {\text{e }} = \, \left( {{\text{c}} + {\text{d}}} \right)/{2}$, ${\text{CHS }} = { 1}00 \, \times { 9}.0 \, /{ 13}.{7 } = { 65}.{7}\quad {\text{e }} = \, \left( {{13}.{9} + {13}.{5}} \right)/{2}$,${\text{CHS }} = { 65}.{7} \quad {\text{e }} = { 13}.{7}$

**Fig. 2:**
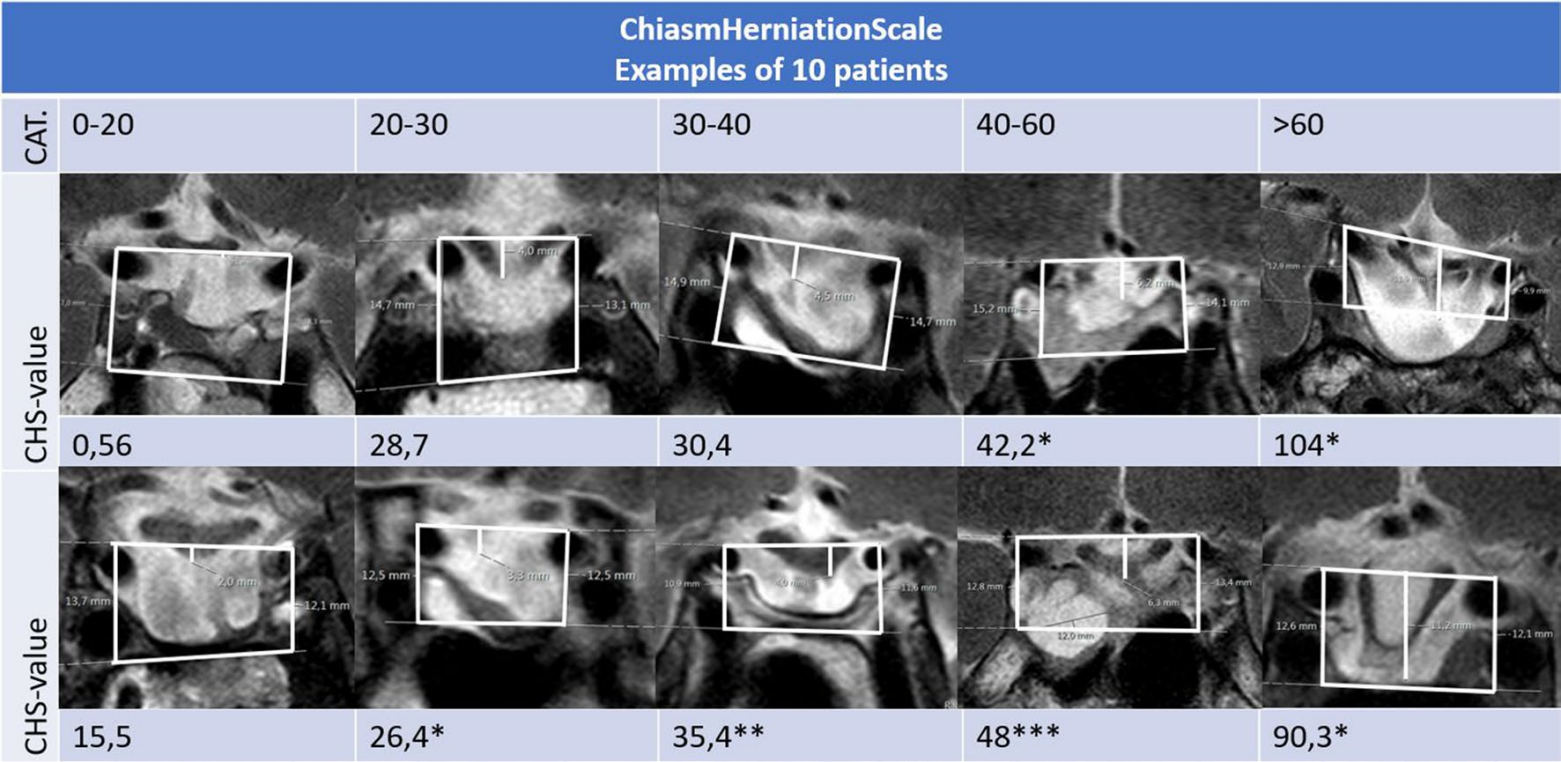
MRI examples of the five categories on the CHS for ten different patients. For each of the five CHS categories two MRI’s are demonstrated with the exact CHS-value below. All MRI’s are obtained from different patients and include the same lines as used in Fig. 1 for calculation of the CHS-value. *With extrapolation of the optic chiasm. **With extrapolation of the line under lower carotid arteries. ***In the left lower corner, a tumour residual is visible. There is no compression on the optic chiasm. *CAT* category

Secondly, all cases were classified using the newly, by our team developed, CHS (M.T., W.R.F., M.C.K., I.C.M.P.), as depicted in Fig. 1. Authors were blinded for visual outcomes. This scale uses the proportional downward displacement of the optic chiasm compared to the sella turcica and the carotid arteries. These two structures, the sella and the carotid arteries, are closely related in a for the rest anatomic variable region. In this radiologic scale the most posterior coronal slice depicting a complete cross sectional view of the carotid arteries in segment C4 and C6 as defined by Bouthilier, is used [34]. The upper boundary, connecting the two carotid arteries in C6 and the lower boundary connecting the two carotid arteries in C4 are drawn as depicted in Fig. 1, line a and line b. The length of these horizontal lines has no influence on the final outcome. Next, the herniation in mm is defined as the distance between the lowest point of the optic chiasm, or visual system, to the upper horizontal line. Sometimes the lowest point can be identified in a different slice and has to be extrapolated. Afterwards this herniation (in mm) is divided by the mean distance (in mm) between the upper and lower boundaries and multiplied by 100 to create the CHS. An example of the calculation of the CHS is depicted in Fig. 1. Based on the CHS, patients were divided into 5 categories: CHS < 0, CHS 0–20, CHS 20–40, CHS 40–60 and CHS > 60, for which examples are shown in Fig. 2.

In ten random patients, radiographs were scored three times to calculate intra- and inter-observer variability (M.T., I.C.M.P.). The intra-class coefficient (ICC) was 0.961 (95%CI 0.853–0.990) for the intra-observer variability, as calculated using reliability analysis. The ICC was 0.914 (95%CI 0.676–0.978) for the inter-observer variability.

In addition, we developed a simplified scale, using the distance between the lowest point of the visual system and the upper boundary connecting the carotid arteries in segment C6 (line g). There is a significant relation between the CHS and this simplified CHS (r = 0.956, p = 0,000).

### Statistics

Data is presented as number (%), mean (± SD), median (IQR) or regression coefficient (95% confidence interval). For measurements in MRI scans we used Sectra Workstation, IDS7 (version 20.2, Sectra AB©, Linköping, Sweden). All data was analysed using SPSS statistical software package (version 23, IBM Corp.©). To account for the different amounts and times of measurements and the relationship between two eyes, a generalized linear mixed model was applied. Onset of herniation and of MD deterioration was analysed using a survival analysis with Kaplan Meijer curves and Log Rank (Mantel-Cox) for comparison. For improvement between MD pre-treatment, best achieved MD after treatment and last measured MD an ANOVA-test (Tamhane’s) has been applied. For the relation between the CHS and the MD both continuous data and categorial data have been used. A p-value less than 0.05 was considered statically significant.

## Results

### Patient characteristics

**Fig. 3:**
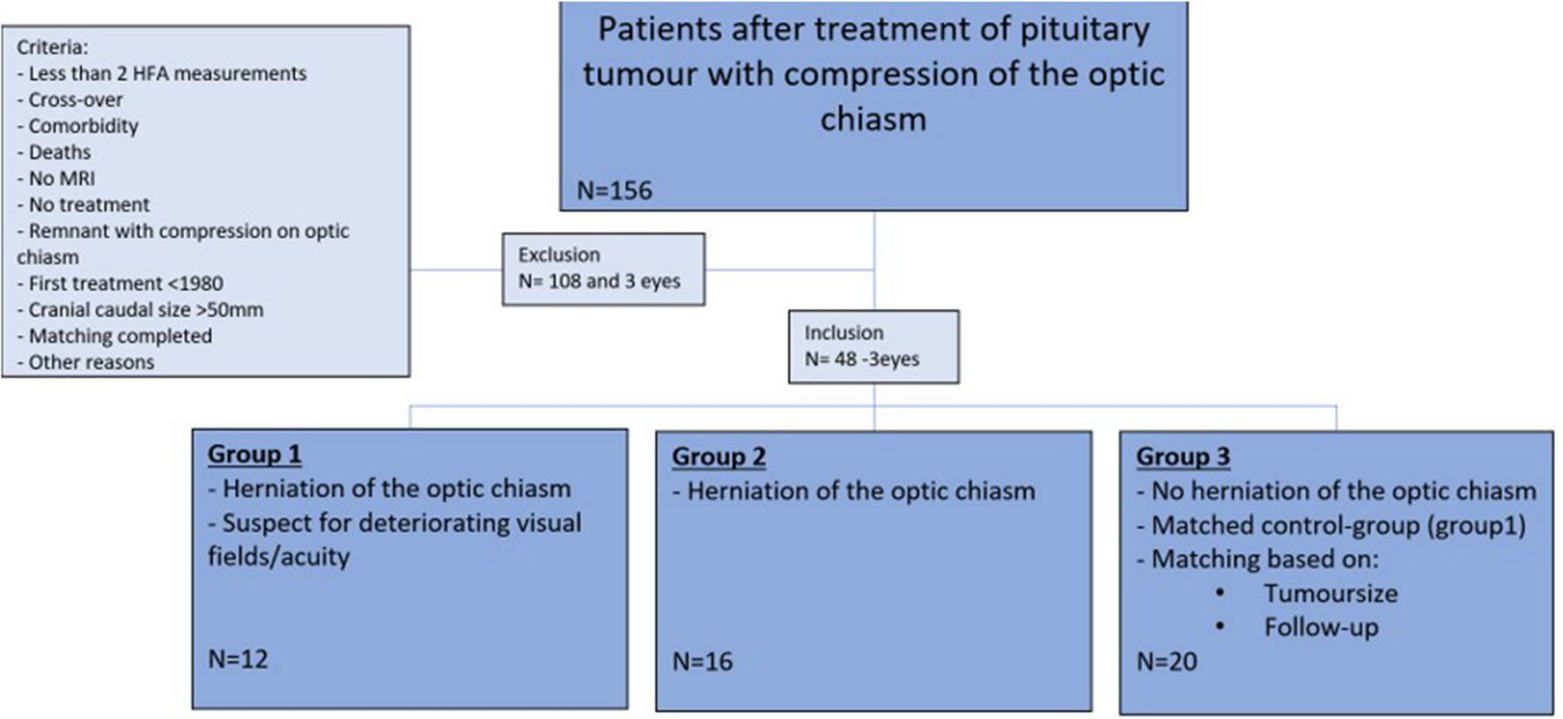
Flowchart of inclusion. From 156 patients, 48 patients are included into three groups, and from 3 of these included patients only 1 eye is included. Group 1 matches with Group 3 based on tumour size and follow-up, 20 patients are excluded because matching was complete

Sixty-seven patients with pituitary macroadenoma, presented to the department of Ophthalmology or Endocrinology of the LUMC between 1985 and 2019, were included, as is shown in Fig. 3. After data collection, 16 patients and 3 eyes were excluded based on comorbidity, absence of at least 2 HFA VF analysis measurements, cross over, or recurrence of tumour growth with chiasmal compression. Moreover, three patients in Group 1 had other deviations of the optic chiasm [horizontally twisted optic chiasm due to scar tissue (N = 1) and atrophic optic chiasm with enlarged empty sella (N = 2)], however they did not have a downward displaced optic chiasm and were therefore excluded. No patients had a primary empty sella. Ultimately, 48 patients were included in the analysis and 19 patients and 3 eyes were excluded (Fig. 3).

Baseline characteristics of the included 48 patients (Gr1 = 12 Gr2 = 16, Gr3 = 20) are depicted in Table 1. The matching process was successful, resulting in no significant differences in tumour size and follow-up duration between the three patient groups. By contrast, age at the start of first treatment differed between the three groups, with Group 3 being significantly older than Group 1. Furthermore, tumour type differed between the groups, since Group 1 was comprised of less patients with NFAs and more patients with prolactinomas and RCCs. As expected, the CHS differed between the patient groups, since Group 3, the group without herniation, scored significant lower (CHS = 8) compared to the groups with herniation, Group 1 and Group 2 (CHS = 63 and CHS = 57, respectively, p < 0.0001).

**Table 1:** Patient characteristics

Clinical characteristics	Group 1 N = 12	Group 2 N = 16	Group 3 N = 20	p value
Sex (male)	6 (50%)	6 (38%)	11 (55%)	0.572
Age at the start of first treatment (years)	44 (± 14) 0.037^¥^	49 (± 12)	58 (± 15)	0.020
Tumour size (mm)*	27 (± 7.8)	27 (± 7.0)	26 (± 9.9)	0.954
Fujimoto score**	3.5 (± 0.53) 0.032^¥^	2.36 (± 1.29) 0.028†	2.33 (± 1.23)	0.058
Follow-up time (years)	15 (± 9.7)	13 (± 7.8)	11 (± 7.4)	0.443
Non-functioning adenoma (NFA)	6 (50%) 0.003	13 (81%)	19 (95%)	0.010
Surgery	10 (83%)	16 (100%)	20 (100%)	0.044***
No of operations per patient	1.25 (± 0.87)	1.31 (± 0.60)	1.05 (± 0.22)	0.363
Radiotherapy	1 (8.3%)	5 (31.3%)	4 (12.5%)	0.333
Chiasm herniation scale (CHS)	63 (± 30.8) 0.000^¥^	57 (± 29.2)	8.3 (± 9.8) 0.000^§^	0.000
No of eyes with MD deterioration	16/24 (67%) 0.002^¥^	9/32 (28%) 0.004†	10/37**** (27%)	0.002
Pre-treatment MD in dB*****	− 6.07 (± 5.89)	− 5.24 (± 5.93)	− 6.23 (± 4.68)	0.799
Pre-treatment VA	0.85 (± 0.19)	0.92 (± 0.24)	0.80 (± 0.30)	0.388

Clinical characteristics of the study population are depicted for the three studied patient groups. The CHS is based on the MRI scan with the most serious herniation. Data are shown as number (%), or mean (SD)

*MD* mean deviation, *No* number, *VA* visual acuity

*Missing values due to missing scan: Gr1: 1, Gr2: 3

**Missing values due to missing pre-treatment MRI scans: Gr1: 4, Gr2: 5, Gr3: 7

***No significant differences between the groups after PostHoc testing

****3eyes excluded

*****Missing values: Gr1: 4, Gr2: 5, Gr3: 4 and 1 eye

^¥^p-value comparing Gr1 and Gr3

^†^p-value comparing Gr1 and Gr2

^§^p-value comparing Gr2 and Gr3

### Improvement of MD after initial treatment in all groups

Prior to treatment, all three groups had similar MDs, reflective of comparable severity in VF defects, as shown in Table 2 (Gr1: − 6.05 dB ± 5.88 Gr2: − 5.24 dB ± 5.93, Gr3: − 6.23 dB ± 4.68, p = 0.799).

**Table 2:** Course of the visual fields around treatment

	Group 1	Group 2	Group 3	p value
Pre-treatment MD***	− 6.06 (± 5.88)	− 5.24 (± 5.93)	− 6.23 (± 4.68)	0.799
Best MD	− 2.74 (± 3.98) 0.103^¥^	− 1.10 (± 2.42) 0.227^†^	− 0.78 (± 2.39)	0.031
Last MD	− 5.14 (± 4.76) 0.089^¥^	− 2.34 (± 3.36) 0.061^†^	− 2.54 (± 3.51)	0.017
Improvement MD pre-treatment to best MD	2.97 (± 6.71)	4.52 (± 5.43)	5.16 (± 4.16)	0.409
Improvement MD pre-treatment to last MD	0.83 (± 6.46)	3.39 (± 6.22)	3.42 (± 4.08)	0.323
Deterioration in dB/month	− 0.003 (95%CI − 0.005 to − 0.001)	− 0.002 (95%CI − 0.005 to + 0.001)	− 0.008 (95%CI − 0.013 to − 0.003)	0.143

*MD* mean deviation

***Missing values: Gr1: 4, Gr2: 5, Gr3: 5

^¥^p-value comparing Gr1 and Gr3

^†^p-value comparing Gr1 and Gr2

^§^p-value comparing Gr2 and Gr3

**Fig. 4:**
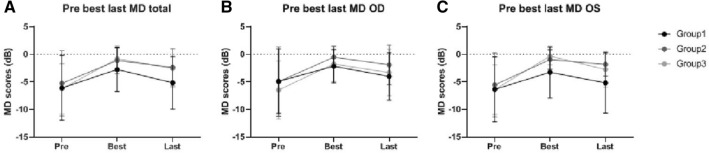
Course of the visual fields around treatment. **a** Shows the MD scores before treatment, best achieved MD scores and latest MD scores for both eyes. **b** Shows the MD scores before treatment, best achieved MD scores and latest MD scores in the right eyes (OD). **c** Shows the MD scores before treatment, best achieved MD scores and latest MD scores in the left eyes (OS)

Comparing the MD scores prior to treatment to the best achieved MD scores following treatment, all groups improved following treatment (Gr1: + 2.97 dB p = 0.097, Gr2: + 4.51 dB p = 0.001, Gr3: + 5.16 dB, p = 0.000), depicted in Table 2 and Fig. 4. However, best MD scores achieved following treatment were significantly different between the groups, with patients in Group 2 and 3 having higher MD scores compared to Group 1 (Gr1: − 2.74 dB ± 3.98, Gr2: − 1.10 dB ± 2.42, Gr3: − 0.78 dB ± 2.39, p = 0.031).

### Onset of visual field deterioration

**Fig. 5:**
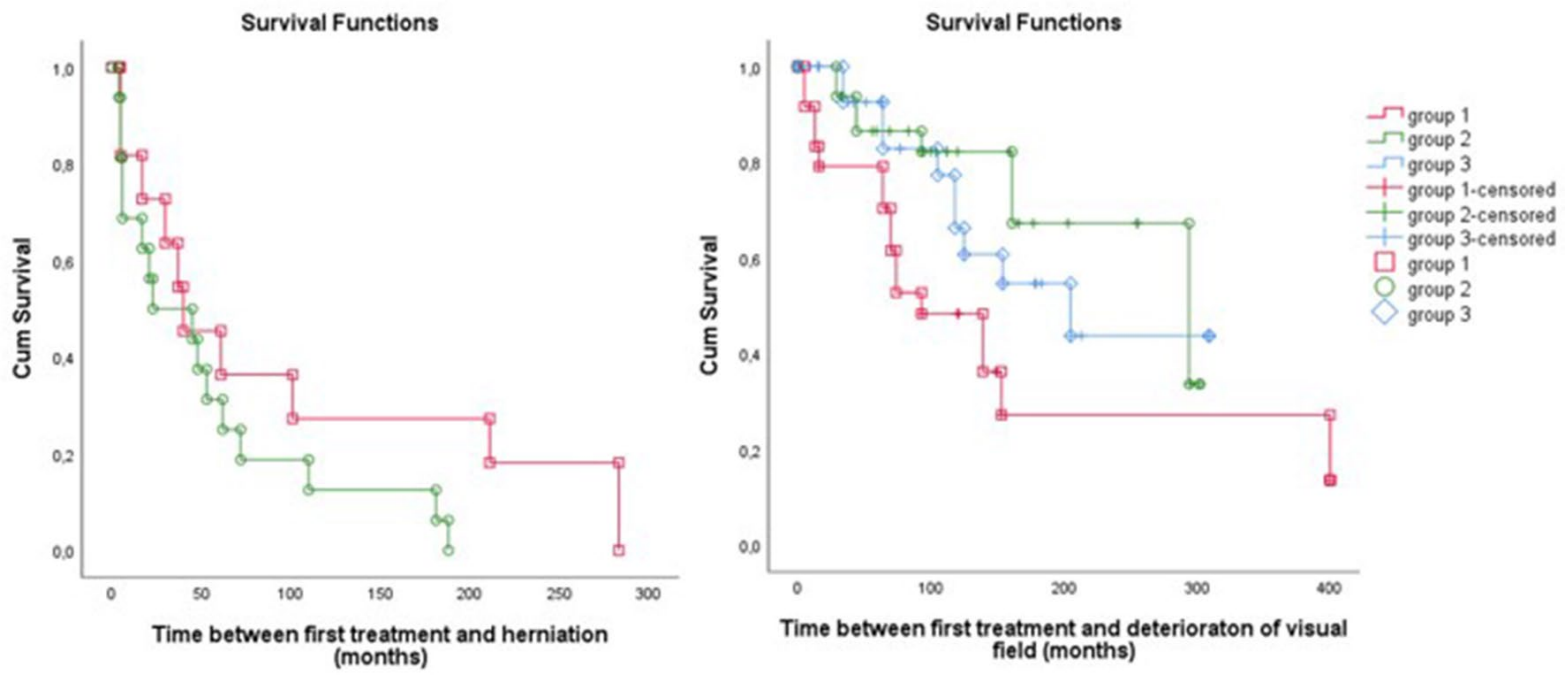
Survival curves of time between treatment and moment of herniation and deterioration. On the left a survival curve showing time between treatment and development of a herniated optic chiasm in Group 1 and 2. On the right a survival curve showing time between treatment and moment of deterioration in Group 1, 2 and 3

After the initial improvement in all groups, some of the eyes deteriorated as shown in Fig. 5. Comparing the median time between treatment and deterioration of the MD, patients in Group 1 exhibited significantly earlier deterioration compared to Group 2 and 3 (Gr1: 93 months Range 5–400, Gr2: 294 months Range 29–302 months, Gr3: 205 months Range 0–309 months, p = 0.022). Moreover, more eyes deteriorated in Group 1 compared to Group 2 and 3 (Gr1 67%, Gr2 28%, Gr3 27%, p = 0.002).

### Onset of herniation of the optic chiasm

After treatment, the time until development of a herniation of the optic chiasm is shown in the Survival curve in Fig. 5. For both Groups 1 and 2 together, the median was 40 months (IQR 6 months to 10 years). There was no significant difference in the moment of onset of herniation between the two groups (p = 0,172).

### No relation between herniation and deterioration of visual fields

**Fig. 6:**
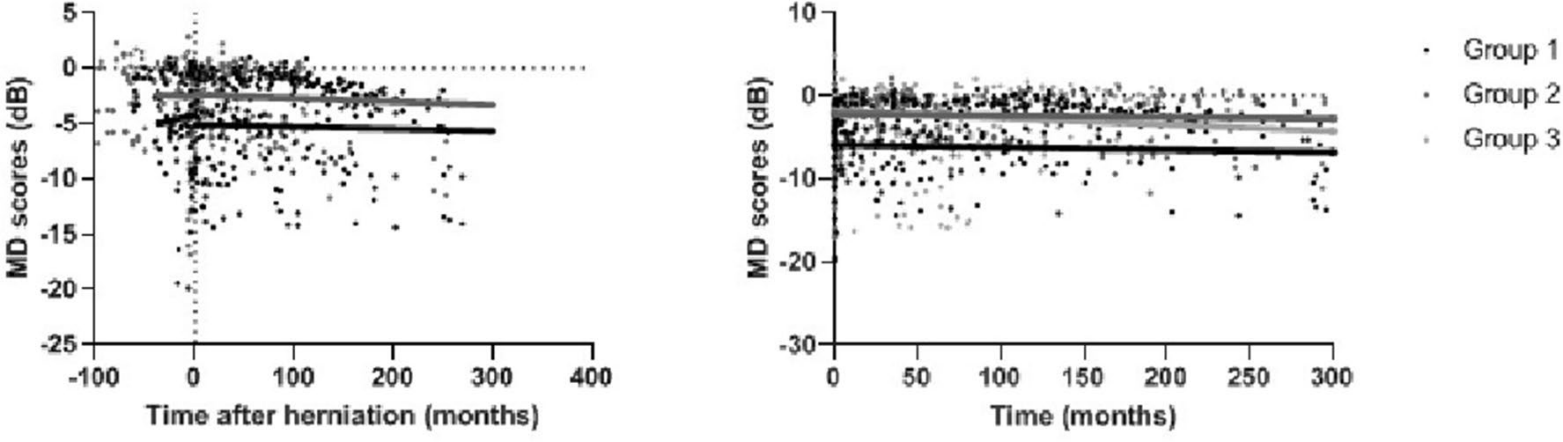
Course of visual fields. On the left the course of the VF around the moment of herniation (time = 0) for Group 1 and 2. On the right the long-term course of VF after treatment for Group 1, 2 and 3

Changes in the course of MD deterioration around the moment of herniation are shown in Fig. 6. There was no significant difference between Group 1 and Group 2 (before p = 0.297, after p = 0.885). In the time between treatment and herniation, a slight improvement of MD is visible (+ 0.006 dB/month, 95%CI − 0.004 to 0.017) and after herniation a slow deterioration is visible (− 0.002 dB/month, 95%CI − 0.005 to − 0.000). There is no significant difference in the course of the MD before and after herniation (p = 0.237) (Fig. 6).

### Deterioration of visual fields

Following the long-term MD scores after treatment, a slight deterioration over months is visible as depicted in Fig. 6 and Table 2. There is no significant difference in the deterioration of MD between the groups (p = 0.143). The MD scores in Group 1 decreased with − 0.003 dB/month, Group 2 decreased with − 0.002 dB/month and Group 3 decreased with − 0.008 dB/month. However, Group 1, with the lowest best achieved MD score, continued to have a significant lower MD in the follow up (p = 0.011).

Comparing CHS > 60, including 12 patients, with the other categories (< 0, 0–20, 20–40 and 40–60), there is no significant difference (p = 0.172).

### Influencing factors

**Fig. 7:**
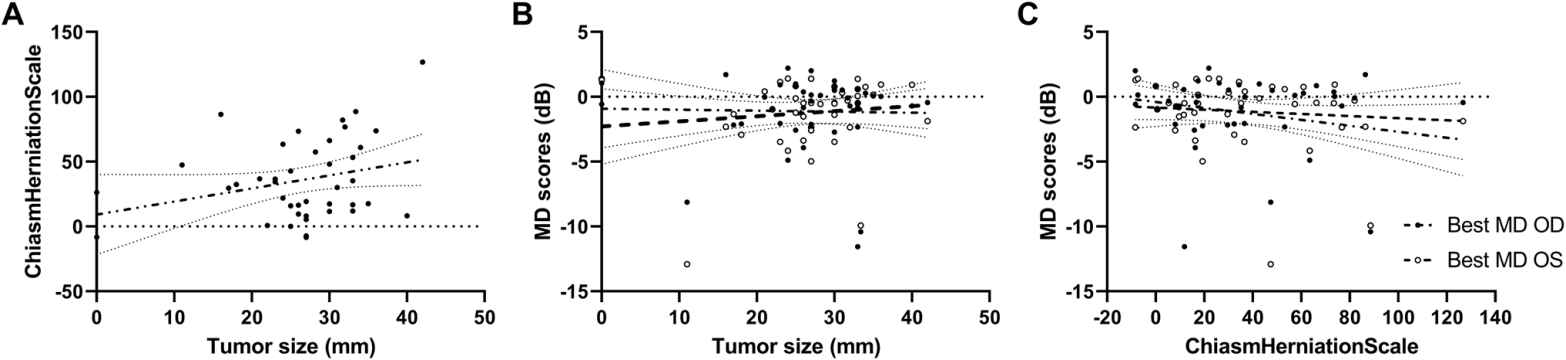
Relation between CHS and tumour size, MD and tumour size and MD and CHS. **a** Shows the relation between the CHS and tumour size, there is a trend to significance (p = 0.084). **b** Shows the relation between the best MD scores and the tumour size. There is no significant relation. **c** Shows the relation between the best MD scores and the CHS. There is no significant relation

Both prior to and following treatment, MD and VA were significantly correlated (before: r = 0.372, p < 0.0001; after r = 0.479 p = 0.000). However, there was no significant difference in VA between the three groups after treatment (p = 0.556), and VA did not influence the course of MD (p = 0.490). Moreover, there was no significant influence of the CHS on the course of MD over time (p = 0.729). Neither did radiotherapy significantly decrease or increase deterioration of VFs in our patients (p = 0.776). Furthermore, there was no relation between the CHS and the best MD (p = 0.496). Neither was there a relation between the tumour size and the best MD (p = 0.623). There was a trend to significance between the tumour size and the CHS (p = 0.084). (Fig. 7).

## Discussion

The aim of this study was to describe the normal course of VF in patients with and without herniation of the optic chiasm after pituitary adenoma treatment and to answer the question whether there is a causal relation between a herniated optic chiasm and the course of the VF. Based on our results, herniation does not influence the course of VF.

In this study, a similar course of the VF, as measured using MD, during long-term follow-up was observed, both in patients with and without herniation of the optic chiasm. Initially, all patients showed improvement after treatment, followed by long-term gradual deterioration. Most patients developed a herniation between 6 months and 10 years after treatment. This time frame was similar to previously reported moment of onset of herniation with 45 published cases, in which patients developed herniations between 1 day and 14 years after treatment [2, 9, 10, 13–22, 24–30]. There was no significant difference in the VFs before and after herniation of the optic chiasm. Chiasmal herniation does not appear to influence the outcome of VF defects in patients with pituitary macroadenomas.

Previously mentioned literature, consisting of 45 patients in 23 studies, shows large discrepancies. There are many contradicting outcomes and there is no evidence-based treatment of a herniated optic chiasm. Our results correspond with the largest case–control study to date, which included eight patients with secondary empty sellae and herniation of the optic chiasm. The authors concluded that herniation leads to marginal to no visual symptomatology, and that severity of visual symptoms and degree of herniation were not correlated [10]. Two other studies correspond with our results, including two patients where no treatment was given and VF/VA remained stable for over 1 year [26, 32].

In eighteen cases, resurgery (e.g. chiasmapexy) was performed resulting in fourteen cases with VF/VA improvement [9, 14, 16, 17, 19, 20, 23–25, 28, 29, 35]. In two cases, VF and VA did not change and in one case VF further deteriorated [18]. Different types of operations were performed including craniotomy, intra-/extradural chiasmapexy with ‘packing’ of the empty sellae with different types of packing material and untethering of scar tissue. In one case, VF/VA improved spontaneously after a light head trauma [9].

Moreover, in fifteen cases treated with withdrawal/reduction of dopamine-agonist, VF/VA improved, but the herniation did not change, which implies the absence of causality [2, 13, 14, 22, 27, 28, 30, 36]. The restart of a dopamine-agonist in one case did not improve vision, nor affect the herniation [21].

Although several cases of successful chiasmapexy are described in international literature, suggesting that herniation of the optic chiasm was the problem, the question why a chiasmapexy did help these patients remains unsolved. Based on the present study that was not able to provide evidence for a causal relation between radiological herniation and VF deterioration, this procedure is not self-evident. Possible explanations might be publication bias, or the existence of another pathological mechanism. Therefore, further research has to be done to prove the efficacy of this procedure.

Our findings point to the absence of a causal relationship between herniation of the optic chiasm and deterioration of VF, since no relation between herniation and deterioration of VF was found, neither on the long-term, nor around the moment of herniation. Furthermore, patients in Group 1, consisting of patients referred for suspicion for deterioration, showed the slightest initial improvement and continued to have significant lower MD values compared to the other patients groups. This difference, not explained by their referral status, could, unfortunately, not be elucidated in this study.

A pathophysiological explanation is that deteriorating VFs are caused by a lesion of the optic chiasm, causing a chiasmal syndrome. The chiasmal syndrome is a constellation of signs and symptoms associated with lesions of the optic chiasm [37]. Different mechanisms can cause lesions of the optic chiasm. We hypothesized that most likely, degree and duration of compression on the optic chiasm before treatment has played an important role. This is supported by the Fujimoto score, a score predicting VFs based on the suprasellar extension of the tumour [38]. In the present study, the Fujimoto score in Group 1 was significantly higher compared to Group 2 and 3 (p = 0.028, p = 0.032). Due to the long follow-up, we did not possess Optical Coherence Tomography (OCT) and Visual Evoked Potential (VEP) data at the start of treatment, but in future studies this might be very interesting. Future studies should investigate factors such as degree and duration of compression on the optic nerves/chiasm and the quality of the optic nerves/chiasm by using MRI, OCT and VEP.

Another explanation might be that tethering of scar tissue has played a role, causing a local lesion of the optic chiasm. This would explain why chiasmapexy, with untethering of scar tissue, has improved VF in some prior published cases.

Based on the patients characteristics, influence of the type of tumour, type of treatment or age cannot be excluded, since Group 1 included significant less NFAs, less operated tumours and younger patients. For example, prolactinomas have been reported more aggressive in male than in female patients [39]. Two male patients with a prolactinoma were included in Group 1, while the other groups did not have any male patient with a prolactinoma. However, excluding these patients from the statistical analysis did not significantly change the results.

More detailed prospective follow-up is needed in a more homogeneous group to understand whether local pressure due to anatomical changes, tethering of scar tissue or other chiasm-related changes induce deterioration of VF. But even in the absence of a causal relation between herniation and deterioration of VF, chiasmpexy might still be useful. However, after the present study it is even more important for surgeons to carefully collect and describe indications and outcome data in case of a chiasmpexy and to design randomized controlled trials.

In the absence of an objective well-defined system to quantify herniation of the optic chiasm, we developed the CHS. Although most studies mention downward displacement of the optic chiasm, only one clear definition has been used in literature. In 1989, herniation was defined by Kaufman et al. as: ‘The anteroinferior third ventricle and part of, or all of, the suprasellar visual system being within the sella turcica; that is, below the theoretical plane of the diaphragm sellae’ [10]. However, this definition does not provide any information on the severity of herniation. Therefore, the newly developed CHS enables to measure the degree of herniation of the optic chiasm on a numeric scale, which allows comparison between patients and to search for a relation between the more severe herniations and VF defects. To prove reproducibility, the ICC for inter- and intra-observer variability have been evaluated and were well over 0.85, respectively 0.914 and 0.961. Limitation of this scale is the use of the Internal Carotid Artery (ICA) segments because of the anatomical variations between patients. However, at this moment, this scale seems to be the best thing there is to measure and compare a herniation of the optic chiasm. In our results, there was no significant relation between the CHS and the MD, the CHS versus the pre-treatment tumour size showed a trend to significance. Additionally, we developed a simplified version of the CHS with a good correlation with the original CHS (p = 0.000), which might be useful in time-sensitive situations or for non-radiologist.

Strong points of this study are the long follow-up, the use of the MD score for VF and the development of the CHS. To the best of our knowledge, this study is the largest study (with 48 patients, 995 MD measurements, and up to 34 years of follow-up) comparing the VF of patients with and without a herniation of the optic chiasm after treatment of a pituitary adenoma. Also, in this study the VFs have been measured with HFA perimetry (MD) instead of VF described by patterns (quadrantopsia, hemianopsia etc.). The HFA perimetry, initially developed for glaucoma patients, allows a more exact outcome and comparison [40, 41]. Recent studies show that this scale can also be used to investigate VFs in patients with pituitary adenomas [33]. Reason to use the MD instead of the VFI was because MD scores have been measured during the whole follow-up period of the patients in this study, where the VFI has been introduced in 2011. Up to now, MD has not really emerged in the field of pituitary outcome research.

Limitations in this study are the absence of good outcome sets for visual outcomes and radiological herniation, and no prospective data or larger scale data. Long-term ophthalmological outcome research in pituitary field has not been well developed and now in the current time of evidence-based medicine and quality of care service evaluations, a good outcome set for visual outcomes needs to be developed. With better prospective data, larger scale data and a good visual outcome set we will be better able to understand deterioration in time in this patient group.

## Conclusion

In conclusion, there is no correlation between visual changes and chiasmal herniation. There is a slow, long-term deterioration of VF over time following treatment-related initial recovery of VF in patients with pituitary macroadenomas. There was neither a difference in the natural course of VF between patients with and without a herniation of the optic chiasm, nor between the course before and after herniation. Post-treatment VFs can most likely be explained by pre-treatment compression of the optic chiasm. In addition, the CHS has been developed to quantify the severity of herniation and although there are some limitations, it is the first quantitative scale that is available for future outcome research. We conclude that herniation of the optic chiasm in this study is merely a radiological feature without apparent consequences for visual function. Based on this data, clinical rationale for surgical intervention for a herniated optic chiasm is not self-evident and needs further study.

## Data Availability

The datasets generated during and/or analysed during the current study are available from the corresponding author on reasonable request.
